# Peroxisome Proliferator-Activated Receptor *δ*: A Target with a Broad Therapeutic Potential for Drug Discovery

**DOI:** 10.1155/2008/736362

**Published:** 2009-04-07

**Authors:** Francine M. Gregoire

**Affiliations:** Department of Biology, Metabolex Inc., Hayward, CA 94545, USA

The biology of Peroxisome proliferator-activated receptor (PPAR) alpha (*α*) and gamma (*γ*) has been intensely scrutinized for the last 20 years and the clinical use of both PPAR-*α* (fibrates) and PPAR-*γ* (thiazolididiones) agonists has led to the understanding of their key role in the treatment of hypertriglyceridemia and type 2 diabetes mellitus [1, 2]. In contrast, the understanding of PPAR delta (*δ*) biology still lags behind. The identification of small molecule agonists for PPAR-*δ* has shed some light on the function of this ubiquitously expressed receptor in preclinical models and early clinical studies [3]. They have revealed the multiple benefits of PPAR-*δ* activation on lipid disorders, diabetes, and inflammation [3, 4]. However, synthetic PPAR-*δ* agonists have yet to be marketed for clinical use in humans, partly due to the burden associated with their clinical development [3].

In this special issue of PPAR Research, the broad potential of PPAR-*δ* agonists for the treatment of metabolic disease is highlighted by 3 key articles. They include a review from de Lange et al. on the regulation of the oxidative capacity of muscle by PPAR-*δ*, an article by Perreault et al. which tackles opportunities and issues with the development of PPAR-*δ* agonist for the treatment of obesity, and finally a review from Wang that addresses the effect of PPAR-*δ* activation on vascular pathophysiological processes. A key question regarding the result of PPAR-*δ* activation, either via natural or via synthetic ligands, is its effect on cell proliferation and the risk of inducing cancer. This has been an area of intense debate as both pro- and antitumorigenic effects have been reported. This topic is concisely reviewed in this issue by Muller et al. Last but not least, two interesting and not well-characterized portions of PPAR-*δ* biology are presented. First, as PPAR-*δ* is expressed at high level in the brain, Hall et al. investigate the potential neuroprotective role of PPAR-*δ* activation in this organ. Second, although the role of PPAR-*δ* in embryo implantation was recognized early on with studies in knockout mice [5], the reproductive functions of PPAR-*δ* are still unclear. This topic and the projected potential applications of PPAR-*δ* ligands in assisted reproductive technology are addressed in Huang’s review.

Taken together, it is obvious that there is an urgent need for additional basic research to better characterize PPAR-*δ* function. The current availability of synthetic ligands should help to further dissect PPAR-*δ*-mediated responses in the brain as well as in other functions not addressed in this issue, including gut and skin homesotasis. Although challenges for the development of PPAR-*δ* agonists remains, they clearly hold great therapeutic promise, as highlighted by recent clinical findings indicating that MBX-8025, one of the most advanced PPAR-*δ* agonists currently in phase II clinical trial for dyslipidemia, displays hypolipidemic features not observed with the currently available dyslipidemia therapies [6, 7].

*Francine M. Gregoire* *Francine M. Gregoire*
